# Molecular evolution and phylogeographic analysis of wheat dwarf virus

**DOI:** 10.3389/fmicb.2024.1314526

**Published:** 2024-02-14

**Authors:** Shiqing Wei, Linwen Liu, Guoliang Chen, Hui Yang, Liang Huang, Guoshu Gong, PeiGao Luo, Min Zhang

**Affiliations:** ^1^College of Agronomy, Sichuan Agricultural University, Chengdu, China; ^2^State Key Laboratory for the Biology of Plant Diseases and Insect Pests, Institute of Plant Protection, Chinese Academy of Agricultural Sciences, Beijing, China

**Keywords:** wheat dwarf virus, viral population history, geography-driven adaptation, phylogeography, Bayesian tip-association significance, nature selection

## Abstract

Wheat dwarf virus (WDV) has caused considerable economic loss in the global production of grain crops. Knowledge of the evolutionary biology and population history of the pathogen remain poorly understood. We performed molecular evolution and worldwide phylodynamic analyses of the virus based on the genes in the protein-coding region of the entire viral genome. Our results showed that host-driven and geography-driven adaptation are major factors that affects the evolution of WDV. Bayesian phylogenetic analysis estimates that the average WDV substitution rate was 4.240 × 10^−4^ substitutions/site/year (95% credibility interval, 2.828 × 10^−4^–5.723 × 10^−4^), and the evolutionary rates of genes encoding proteins with virion-sense transcripts and genes encoding proteins with complementary-sense transcripts were different. The positively selected sites were detected in only two genes encoding proteins with complementary-sense, and WDV-barley are subject to stronger purifying selection than WDV-wheat. The time since the most recent common WDV ancestor was 1746 (95% credibility interval, 1517–1893) CE. Further analyses identified that the WDV-barley population and WDV-wheat population experienced dramatic expansion-decline episodes, and the expansion time of the WDV-barley population was earlier than that of the WDV-wheat population. Our phylogeographic analysis showed that the WDV population originating in Iran was subsequently introduced to Europe, and then spread from Eastern Europe to China.

## Introduction

Wheat dwarf virus (WDV) was first described in the 1960s (Vacke, 1961) and to date it has been considered an important pathogen of economically important cereals, including wheat, barley and oats and widely scattered among European, African and Asian countries (Bisztray et al., 1989; Lindsten and Vacke, 1991; Bendahmane et al., 1995; Kvarnheden et al., 2002; Lemmetty and Huusela-Veistola, 2005; Köklü et al., 2007; Xie et al., 2007; Wang et al., 2008; Liu et al., 2012; Abt et al., 2020). WDV is transmitted via horizontal transmission among plants in a persistent, non-propagative manner by *Psammotettix alienus* (Lindblad and Areno, 2002). This virus invades the midgut of *P. alienus* through receptor mediated endocytosis and spreads through the digestive tract to haemolymph (Wang et al., 2014). However, *P. alienus* does not transmit virions to the eggs and nymphs of the first instar (Vacke, 1964). After infection with WDV, the plants showed streaking, dwarfing of leaves, chlorosis and severe stunting of infected hosts, which resulted in serious crop yield reduction (Vacke, 1972). WDV causes an average yield loss of up to 35% in cereal crops globally, and in some cases up to 90% in localized epidemics (Vacke, 1964; Lindsten and Lindsten, 1999; Lindblad and Waern, 2002; Sirlova et al., 2005).

WDV is a member of the genus *Mastrevirus* in the family *Geminiviridae* (Schubert et al., 2007), which has a circular single-stranded DNA (ssDNA) genome of approximately 2.75 kb (Macdowell et al., 1985). This genome has four open reading frames (ORFs) and encodes four proteins, among which the virion-sense strand encodes movement protein (MP) and coat protein (CP), and the complementary-sense strand encodes two replication-associated proteins (RepA and Rep). When the virion of WDV circulates in the insect vector, the coat protein not only protects the viral genome, but also binds to ARF1 of *P. alienus* to help the virus spread from the gut to the haemolymph (Wang et al., 2019; Bennett and Agbandje-McKenna, 2020). In addition, coat protein facilitates nuclear and cell-to-cell transport of the genome via interaction with the MP and nuclear shuttle protein (NSP) (Macdowell et al., 1985). RepA binds the plant retinoblastoma-related protein pRBR to facilitate the expression of E2F transcription factors and activate genes encoding host DNA replication proteins and accessory factors (Hanley-Bowdoin et al., 2013; Sahu et al., 2013), and both RepA and Rep have RNA silencing suppressor activities (Liu et al., 2014). The WDV genome also contains a long intergenic region (LIR) and a short intergenic region (SIR), which are non-coding sequences that are important for the regulation of viral replication and gene expression (Hofer et al., 1992).

Phylogenetic studies of WDV genome sequences obtained from different host species confirmed that WDV can be divided into two main groups: WDV-wheat and WDV-barley strains (Lindsten and Vacke, 1991; Commandeur and Huth, 1999; Muhire et al., 2013; Schubert et al., 2014). Many experiments showed that WDV-wheat isolates can infect barley plants (Lindsten and Vacke, 1991; Commandeur and Huth, 1999; Kundu et al., 2009; Ramsell et al., 2009; Schubert et al., 2014), but there are many conflicting reports on whether WDV-barley isolates can infect wheat plants. Some field investigations and leafhopper-mediated transmission experiments have shown that the virion from barley plants could not infect wheat plants (Lindsten and Vacke, 1991; Tobias et al., 2011). However, WDV sequences belonging to those two strains were obtained from barley and wheat plants infected with WDV (Wang et al., 2008), and agricultural infecting clones of WDV-barley successfully infected wheat plants (Ramsell et al., 2009).

Although the mutation rate of DNA viruses is not as high as that of RNA viruses, DNA viruses have rapid replication dynamics and large virus populations. Genetic drift, natural selection and gene flow make the evolutionary characteristics and population genetic structure of DNA viruses are still more complicated (Zhan and McDonald, 2004). Stochastic changes in allele frequency in geographical populations lead to the random fixation of neutral alleles, thereby leading to non-adaptive differentiation (Gao et al., 2017). However, an increasing number of reports have shown that viruses have undergone obvious geographical adaptive differentiation via long evolutionary processes (Gao et al., 2017; Mao et al., 2019; Kawakubo et al., 2021). Natural selection is primarily expected to play a central role in the spatial population genetic structure of plant pathogens in agricultural ecosystems (Thrall et al., 2011). Recombination is also an important evolutionary mechanism that produces diversity in most viruses (Pérez-Losada et al., 2015). Recombination is one of the important mechanisms driving WDV population diversity, and extensive intraspecific recombination generally occurs at the boundary of and within the genes encoding Rep and CP. Moreover, SIRs are also hot spots for recombination (Wu et al., 2008, 2015; Parizipouret et al., 2017).

Although there have been many phylogenetic and recombination analyses of WDV (Lindsten and Vacke, 1991; Commandeur and Huth, 1999; Wu et al., 2008; Muhire et al., 2013; Schubert et al., 2014; Wu et al., 2015; Parizipouret et al., 2017), there are few reports on population history and migration patterns on a given time scale. WDV is transmitted by vector insects and its distribution stretches across Eurasia. Whether there are geographical constraints on the evolution of WDV is unknown. In this study, we collected and analysed all of the sequence data from WDV isolates in GenBank to study the temporal dynamic, phylogeographic, and demographic history of WDV.

## Materials and methods

### Dataset

By October 2022, we had searched and obtained all complete genome sequences of each WDV gene from the GenBank database of the National Center for Biotechnology Information. Multiple alignment of nucleotide sequences was conducted with MAFFTv7 software (Katoh and Standley, 2013). To identify potential recombinant sequences of WDV, we calculated for signals of recombination using the RDP 4.95 suite (Martin et al., 2015) with seven software programs, namely, RDP, GENECONV, BOOTSCAN, Maximum Chi-Square (MAXCHI), CHIMAERA, 3SEQ and Sister Scanning (SISCAN). Only the events detected by at least four algorithms were accepted, with an associated *p*-value of 10^−4^. For the accuracy of the results, recombinant isolates were removed and we eventually obtained 315 CP isolates, 299 MP isolates, 294 Rep isolates and 297 RepA isolates for phylogenetic analysis (Supplementary Table S1). Furthermore, we constructed a dataset containing 278 isolates of the whole–genome coding region of WDV.

### Tests for temporal signals

We inferred the evolutionary timescale and substitution rate using a molecular clock calibrated by the sampling times of the sequences in which the temporal signal was evaluated by randomizing the sampling dates over clusters of tips and not over individual tips (Duchêne et al., 2015). The mean substitution rate estimated from the real sampling dates did not overlap with the 95% credibility intervals of rate estimates from 10 replicate datasets with cluster-permuted sampling dates. In addition, we evaluated the time structure of all datasets using newly developed BETS (Duchene et al., 2020). In this method, a heterogeneous model (*M*_het_) and an isochronous model (*M*_iso_) are introduced into the same dataset, and the marginal likelihood values estimated by generalized stepping stone sampling (Baele et al., 2016) are used to compare the fit of two competing data. The best fit model of the dataset was selected by Bayesian factor (BF; Kass and Raftery, 1995), when (log) BF log[P(Y|*M*_het_)] − log[P(Y|*M*_iso_)] is greater than 5, suggesting the presence of a sufficient temporal signal in the data.

### Temporal dynamics of wheat dwarf virus

The substitution models (Supplementary Table S2) of the sequences were selected based on the Bayesian information criterion (BIC) using PartitionFinder (Lanfear et al., 2016) implemented in PhyloSuite (Zhang et al., 2020). Misspecification of the tree prior can lead to incorrect substitution rate (Müller et al., 2018). Therefore, marginal likelihood estimates based on path sampling (Baele et al., 2012) were used in BEAST 1.10 (Suchard et al., 2018) to find the best fit substitution tree prior (including the constant size, exponential growth, and Bayesian skyline coalescent) and best fit substitution clock model (both strict and relaxed clocks). All datasets were using uncorrelated lognormal relaxed clock and Bayesian skyline coalescent tree prior in the subsequent analysis (Supplementary Table S3). The Markov chain Monte Carlo (MCMC) analyses were run for 500 million generations, with sampling every 25,000 steps. Sufficient sampling was assessed by estimating the effective sample sizes values of all parameters and by inspecting traces implemented in Tracer 1.7 (Rambaut et al., 2018), and first 10% of the samples were discarded as burn-in.

### Discrete phylogeographic analyses

An asymmetric substitution model with Bayesian random search variable selection options was implemented in BEAST based on the obtained optimal substitution rates for the whole–genome coding region (Supplementary Table S4), and Bayes factor (BF) were used to estimate significant diffusion rates between any two positioning states in 7 geographic regions, and the rates of viral migration across the discrete locations were calculated from the resulting log file in SpreaD3 (Bielejec et al., 2016). Significant migration pathways were determined based on the criteria of a BF > 3 and a mean indicator and mean indicator >0.5. The categories of rate support were as follows: decisively supported diffusion, BF > 1,000; very strongly supported diffusion, 1,000>BF ≥150; strongly supported diffusion, 150 >BF ≥ 10; and supported diffusion, 10 >BF ≥ 3 (Su et al., 2015).

### Phylogeny-geography association and population structure analyses

A method accounting for phylogenetic uncertainty in investigating phylogeny-trait correlations with 1,000 random permutations of tip locations was implemented with BaTS 2.0 software to estimate the null distribution for each statistic (Parker et al., 2008); this was used to calculate three summary statistics (association index, *AI*; parsimony score, *PS*; and monophyletic clade, *MC*) from the posterior sample of trees produced with BEAST1.10 (Suchard et al., 2018) to identify the potential effects between the phylogeny and the geographic structure of WDV which the isolates only from wheat plant. The *MC* index assesses the association of geographic origin to phylogeny, and the *AI* and *PS* assess the association between trait and tree topology. High *MC* scores and low *AI* index and *PS* values identified a strong phylogenetic trait association and low spatial admixture. In addition, we investigated the geographic regions of WDV using the discriminant analysis of principal components (DAPC) method independent of Hardy–Weinberg equilibrium and panmixia (Jombart et al., 2010).

### Analysis of selection pressures

To investigate the difference in selection pressure between WDV-wheat and WDV-barley, we used the branch and site-specific models (Yang and Nielsen, 1998; Yang and Nielsen, 2002) of the CODEML algorithm (Yang, 2007) implemented in EasyCodeML (Gao et al., 2019). There are two models were used in the branch models, a ratio model assumed the ω-value for the entire tree, and a two-ratio model allowed the ω-value to vary between the two strains. For the site-specific models, three different sets of nested models (M3 vs. M0, M2a vs. M1a, and M8 vs. M7) were compared, and likelihood-ratio tests (LRTs) were applied to select the one that best fit the data. When the LRT was significant (*p* < 0.01), we used the Bayes empirical Bayes (BEB) method (Yang et al., 2005) to identify amino acid residues that four genes, CP, MP, Rep and RepA, are likely to have evolved under positive selection (posterior probability >0.95).

## Results

The datasets containing all WDV-barley and WDV-wheat isolates and the datasets containing only WDV-wheat isolates passed the DRT, but the datasets containing only WDV-barley isolates did not pass the DRT (Supplementary Figure S1). Further BETS analysis results showed that the log-marginal likelihood value of *M*_het_ for all datasets was higher than the log-marginal likelihood value of *M*_iso_ (Supplementary Figure S1), indicating that that our datasets had sufficient time signals for reliable Bayesian tip analysis.

### Evolutionary rates and timescales

Bayesian skyline coalescent tree priors and uncorrelated lognormal relaxed clocks provided the best fit to all of the datasets (Supplementary Table S3). We calculated the MRCA and evolutionary rate of the WDV CP, MP, Rep and RepA genes and WDV genome-wide coding region using five datasets. In addition, the rate of evolution and the MRCA of WDV-wheat and WDV-barley strains were also examined for each gene and for the genome-wide coding region. Our results showed that the evolutionary rate varied greatly among genes. The evolutionary rate of CP was 5.985 × 10^−4^ substitutions/site/year (95% credibility interval: 3.970 × 10^−4^–8.243 × 10^−4^), and the evolutionary rate of MP (1.309 × 10^−3^ substitutions/site/year, 95% credibility interval: 7.117 × 10^−4^–1.993 × 10^−3^) was more than twice that of CP (Supplementary Table S4). Although both the Rep gene and RepA gene (8.149 × 10^−4^ substitutions/site/year, 95% credibility interval: 5.937 × 10^−4^–1.065 × 10^−3^) encoded replication-related proteins, the evolutionary rate of RepA was significantly faster than that of Rep (Supplementary Table S4). The TMRCA obtained for different coding regions varied, and the TMRCA obtained from the whole–genome coding region of WDV was 1746 CE (95% credibility interval: 1517 CE-1893 CE, Supplementary Table S4). The TMRCA obtained for CP, MP, Rep and RepA was 1837 CE (95% credibility interval: 1662–1939), 1885 CE (95% credibility interval: 1746–1980), 1897 CE (95% credibility interval: 1781–1969) and 1921 CE (95% credibility interval: 1831–1974), respectively (Supplementary Table S4). In addition, to assess the differences in evolutionary rates between WDV-wheat and WDV-barley, the existing 5 datasets were divided again according to strains to obtain a total of 10 datasets. These datasets were calibrated according to the evolutionary rates obtained from the datasets that passed the DRT, and the evolutionary rate of WDV-wheat was faster than that of WDV-barley, as measured by CP, Rep, RepA and the whole–genome coding region of WDV (Supplementary Table S4). Surprisingly, the evolutionary rate of MP in WDV-barley was significantly faster than that in WDV-wheat. However, the results obtained from the datasets of either coding region showed that the barley strain of WDV had an earlier TMRCA than the wheat strain.

**Figure 1:**
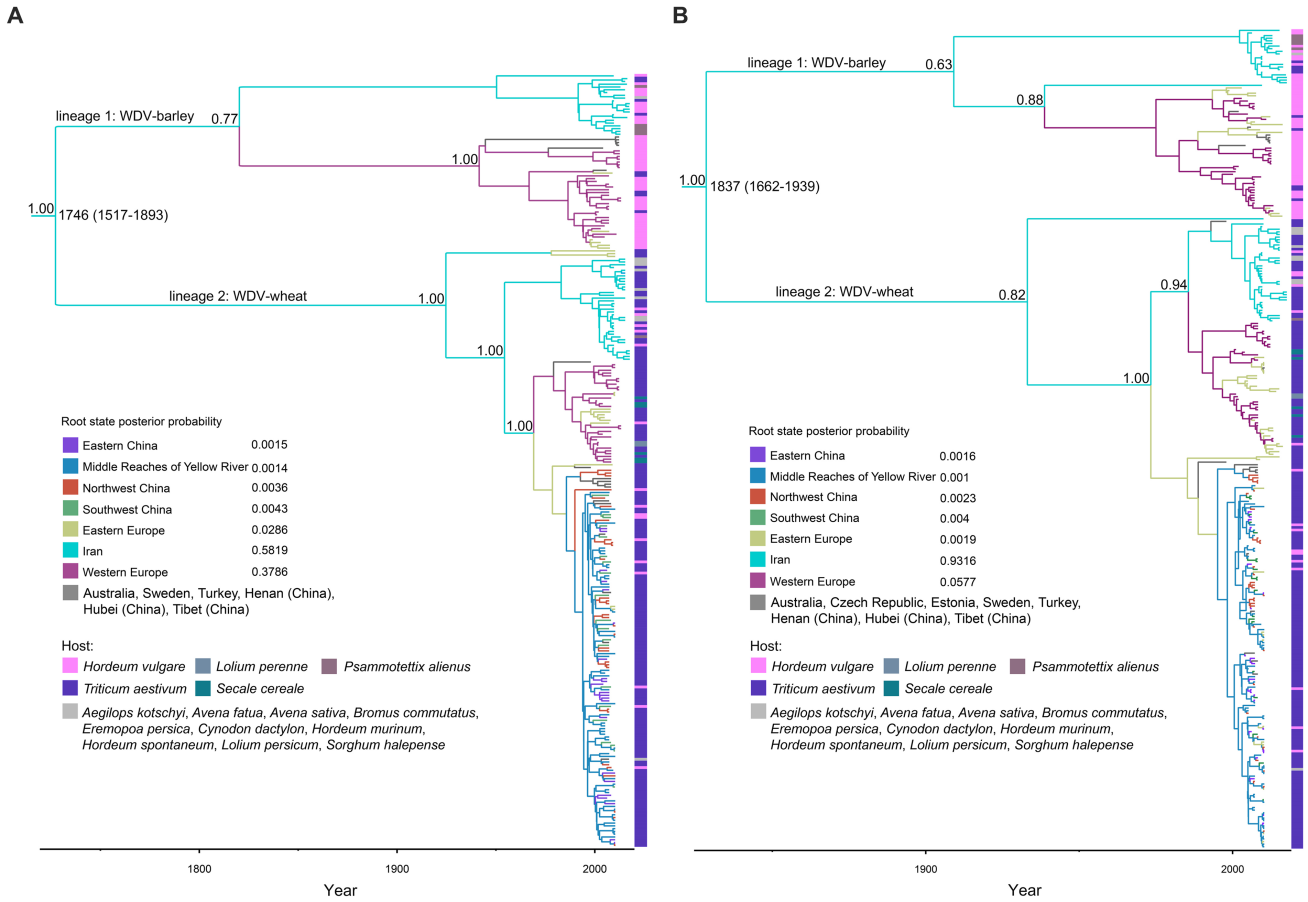
Time-scaled maximum clade credibility tree inferred from the whole–genome coding region **(A)** and coat protein **(B)** sequences of wheat dwarf virus. The tree topologies have been chosen to maximize the product of node posterior probabilities. Branch lengths are scaled according to time, as shown by the horizontal axis. Branch colors represent inferred location states. The inset panel is the root state posterior probabilities of the geographic regions.

The maximum clade credibility (MCC) trees inferred from the whole–genome coding region and the CP datasets shared very similar topologies, and all isolates could be separated into two major clades WDV-wheat and WDV-barley (Figures 1A,B). In both wheat and barley strains, isolates from Iran were clustered on one branch. Our Bayesian analysis placed the root of the trees for Iranian isolates with a higher posterior probability than for isolates from other regions, which was supported by two datasets: one dataset containing the WDV coat protein coding region and another dataset containing the WDV whole–genome coding region (Figure 1).

### Global migration pattern of wheat dwarf virus

**Figure 2:**
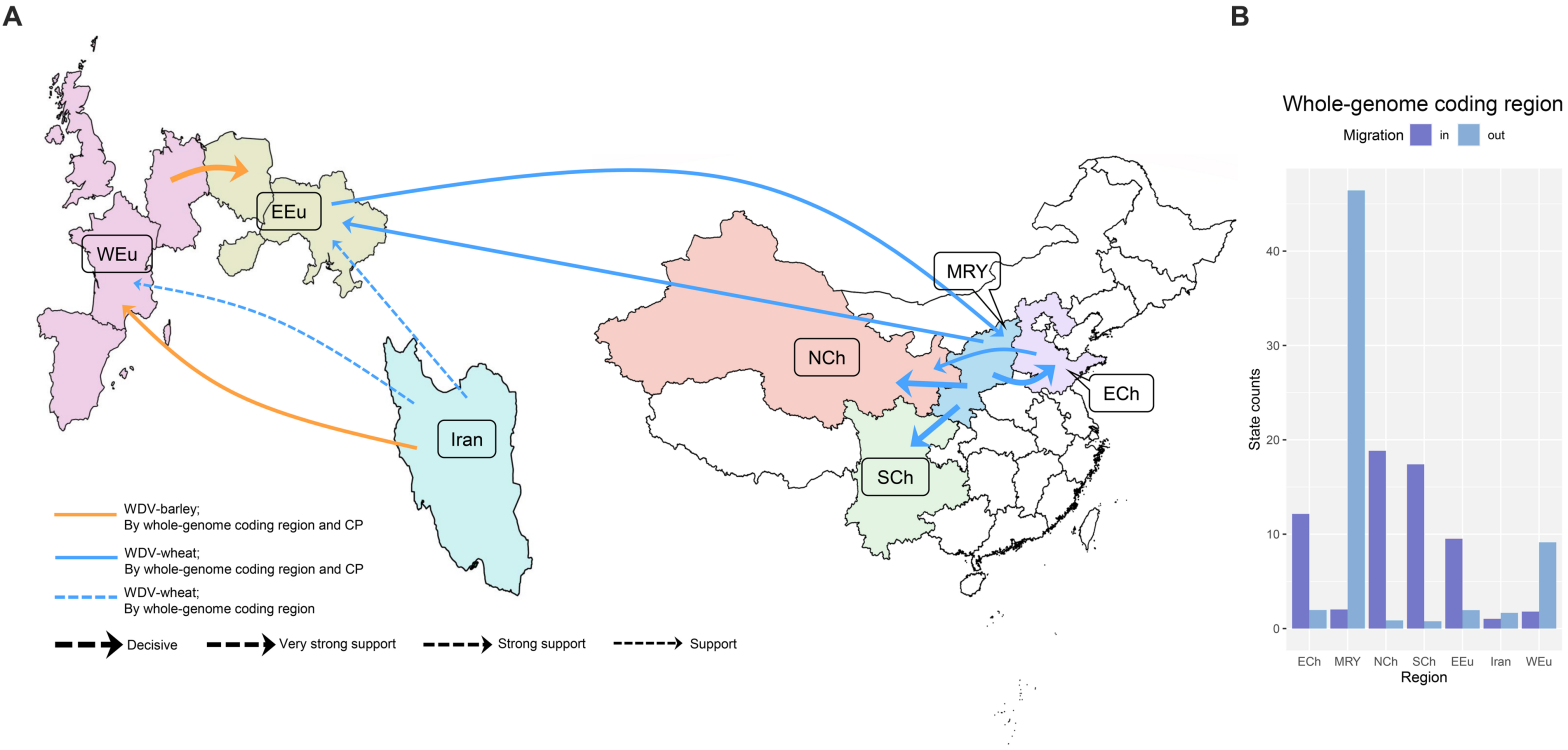
The spatial dynamics analysis of wheat dwarf virus inferred from whole–genome coding region and coat protein. **(A)** Supported global spatial diffusion pathways and **(B)** histogram of the total number of location-state transitions inferred from whole genome coding region. ECh, Eastern China; EEu, Eastern Europe; MRY, Middle Reaches of Yellow River; NCh, Northwest China; SCh, Southwest China; WEu, Western Europe.

Ten migration pathways within the spatial diffusion of WDV were supported by our Bayesian phylogeographic analysis based on the whole–genome coding region datasets, eight of which were supported by WDV-wheat dataset (Figure 2; Supplementary Table S5). There are four migration pathways from the middle reaches of the Yellow River (Shanxi and Shaanxi provinces) to Eastern Europe (Hungary, Poland, and Ukraine), Eastern China (Hebei and Shandong provinces), Northwest China (Gansu, Ningxia, Qinghai and Xinjiang provinces) and Southwest China (Sichuan, Yunnan and Guizhou provinces) supported by WDV-wheat dataset. There was also a migration pathway from China and from Eastern China to Northwest China supported by WDV-wheat dataset. There were two migration pathways from Iran spread to Western Europe (France, Germany, Spain, and UK) and Eastern Europe, and one migration pathway from Eastern Europe to the middle reaches of the Yellow River supported by WDV-wheat dataset (Figure 2; Supplementary Table S5). Moreover, there were two migration pathways from Iran and Western Europe, which spread to Western Europe and Eastern Europe, respectively, supported by WDV-barley dataset (Figure 2; Supplementary Table S5).

Since the dataset of CP gene contained more isolates from Hungary, we further analysed the global migration pathways of WDV using CP gene. Not surprisingly, the results that we obtained were similar to those obtained from the whole–genome coding region of WDV, except for the migration pathway from Eastern China to Northwest China (Supplementary Table S5).

The inferred spatial dynamics of WDV suggest that Western Europe and the middle reaches of the Yellow River have acted as important sources for epidemics emerging in other regions. This was also supported by the state change counts, with migration from the middle reaches of the Yellow River and Western Europe being much greater than that from any other geographic region included in our analysis (Figure 2). Eastern Europe, Eastern China, Northwest China and Southwest China are the main immigration regions (Figure 2).

### Spatial dynamics of wheat dwarf virus over time

**Figure 3:**
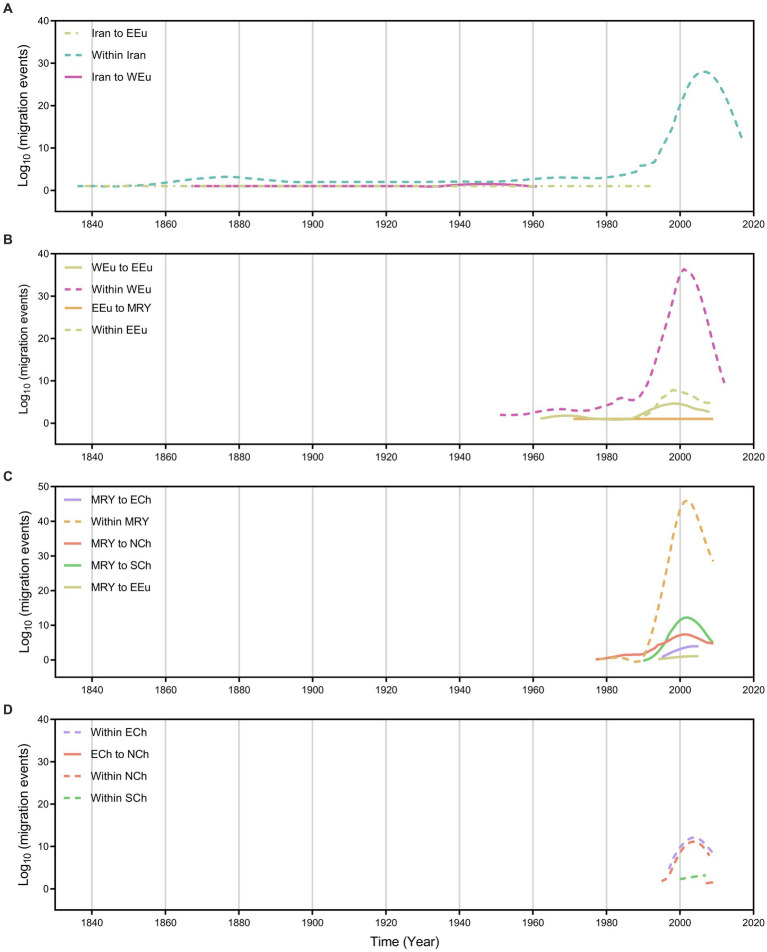
Inferred migration events (on log_10_ scale) of wheat dwarf virus over time (year) with each region. **(A)** Migration events from and within Iran, **(B)** Migration events from and within EEu and WEu, **(C)** Migration events from and within MRY, **(D)** Migration events from ECh and within ECh, NCh and SCh. ECh, Eastern China; EEu, Eastern Europe; MRY, Middle Reaches of Yellow River; NCh, Northwest China; SCh, Southwest China; WEu, Western Europe.

We further summarize the WDV migration load and direction across time (Figure 3). The first wave of WDV emigrations from Iran to Eastern Europe had started before the 1840s and stopped in the early 1990s, the second wave of WDV emigrations also from Iran and spread to Western Europe had started after the 1860s and the migration stopped approximately in the 1960s (Figure 3A). However, the migrations from Western Europe to Eastern Europe, from Eastern Europe to the middle reaches of the Yellow River were not started until after the 1960s (Figure 3B). The migrations of the middle reaches of the Yellow River to Northwest China had started in the late 1970s, and the migrations of the middle reaches of the Yellow River to Eastern Europe, East China, and Southwest China began in the 1990s and began to decline in the late 2010s (Figure 3C). In addition, there was a brief migration of Eastern China to Northwest China in the 2010s (Figure 3D).

### Demographic history of wheat dwarf virus

**Figure 4:**
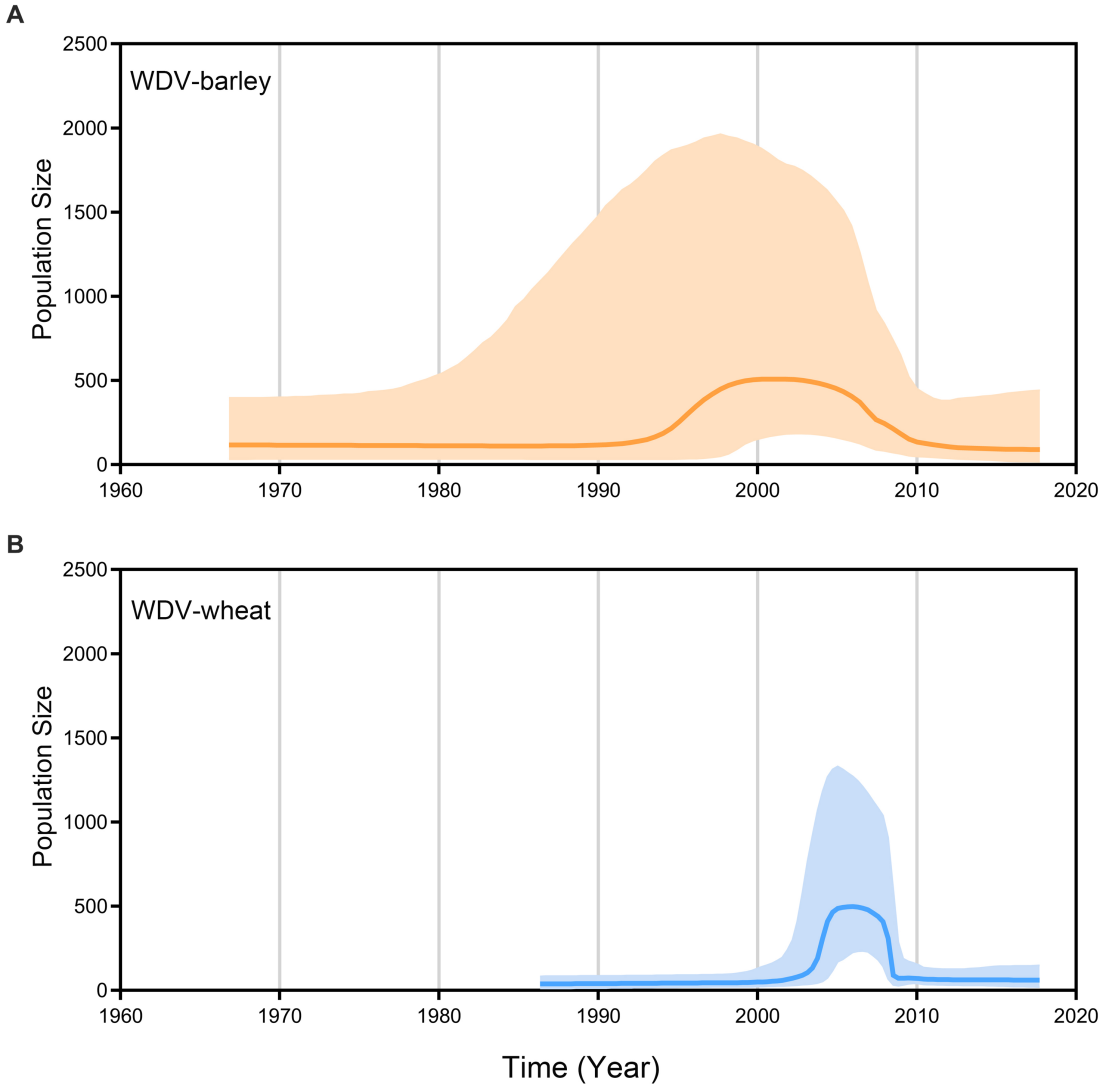
Bayesian skyline plots of population dynamics for barley strain **(A)** and wheat strain **(B)** of wheat dwarf virus inferred from whole–genome coding region. The *x*–axis represents units of years whereas the *y*-axis gives the population size (net).

A coalescence-based BSP generated by the whole–genome coding region data revealed the explicit demographic history of WDV, showing that WDV populations experienced a dramatic expansion in the early 1990s and then began to decline sharply for approximately 15 years (Figure 4). Our results show that the populations of WDV strains in barley and wheat remained stable until 1990, but it is clear that the population size of WDV-wheat is smaller than that of WDV-barley, and that WDV-wheat emerged later than WDV-barley. The population size of WDV-barley experienced expansion for approximately 7 years from the early 1990s to the 2000s, followed by population decline in approximately 2005. However, the population size of WDV-wheat began to expand in approximately 3 years in approximately 2003, began to decline sharply in less than 3 years in approximately 2007, and subsequently became consistent with the population size of WDV-barley (Figure 4).

### Geographic structure patterns of wheat dwarf virus

The strain structure of WDV was apparent in the maximum clade credibility (MCC) trees and phylogenetic trees (Figure 1), but the geographical structure was not evident. Therefore, we investigated the global trait association of phylogeography structure with the evolution of WDV (*p*_AI_ < 0.001 and *p*_PS_ < 0.001, Table. 1). According to the *MC* statistic for single genes and whole–genome coding region of WDV, there were significant population segmentation was observed for the 4 geographic regions (Table 1). The DAPC scatter plots indicated that the population from Iran was relatively distinct from the other populations along the first discriminant function axis (Supplementary Figure S2). The isolates from Europe are clearly divided into groups: Eastern and Western European, while all isolates from China are significantly clustered together and separated from the groups of Eastern and Western Europe on the second discriminant axis (Supplementary Figure S2). In addition, we conducted the global trait association of phylogeography structure and PCA analysis of the four WDV coding genes respectively, our results showed that the differentiation of the four WDV coding genes had strong geographical correlation (Supplementary Table S6; Supplementary Figures S3, S4).

**Table 1:** Analysis of the phylogeographic structure of wheat dwarf virus.

Coding region analysed	Statistic	Isolates	Observed mean (95% HPD CIs)	Null mean (95% HPD CIs)	*p*-value
Whole–genome coding region	AI		3.940 (3.219–4.658)	11.664 (10.583–12.729)	<0.001
	PS		31.132 (29.000–33.000)	61.153 (58.783–63.276)	<0.001
	MC (China)	114	4.349 (4.000–5.000)	1.290 (1.000–2.000)	<0.01
	MC (Eastern Europe)	14	5.698 (5.000–9.000)	1.686 (1.052–2.114)	<0.01
	MC (Iran)	27	17.060 (14.000–19.000)	5.870 (4.412–9.005)	<0.01
	MC (Western Europe)	24	3.094 (3.000–4.000)	1.853 (1.160–2.463)	<0.02

### Selection pressure analysis of wheat dwarf vieus

**Figure 5:**
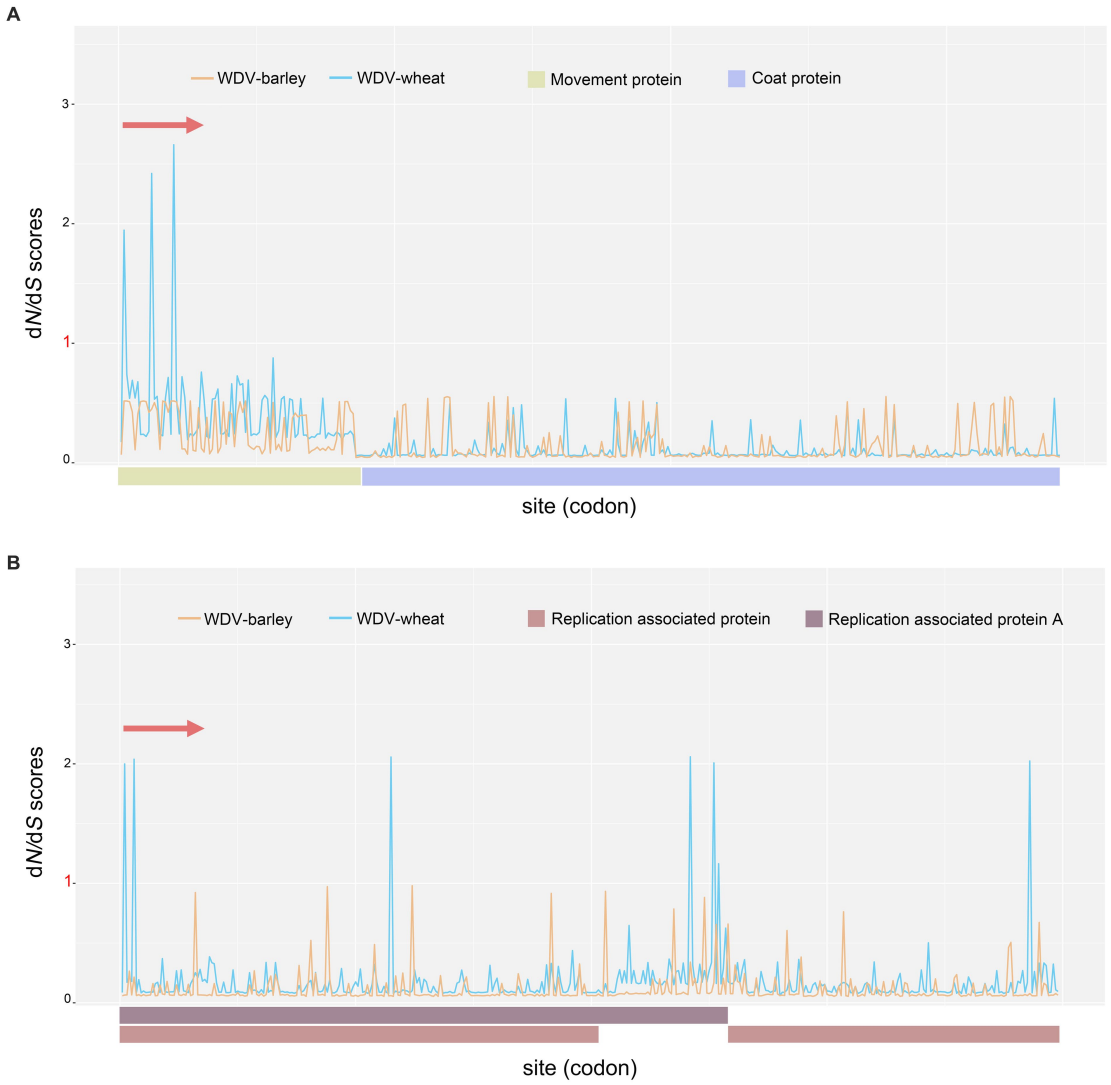
The *d*N/*d*S ratios for the virion-sense strand coding region (CP and MP genes; **A**) and the complementary-sense strand coding region (Rep and RepA genes; **B**) of wheat dwarf virus.

To analyse possible selection pressure in the WDV coding region, the ratio of nonsynonymous to synonymous sites (*d*N/*d*S) was calculated. Our results indicated that negative selection was acting on the WDV whole–genome coding regions, and purifying selection was the main evolutionary force (Supplementary Table S7). Among the four protein-coding genes, the CP gene was subjected to the strongest selection pressure (Supplementary Table S7), and for the four protein-coding genes of WDV, WDV-barley was subjected to stronger purifying selection than WDV-wheat (Figure 5; Supplementary Table S7). Moreover, five positively selected sites were detected in two genes encoding replication proteins in WDV-wheat (Supplementary Table S8). Three positively selected sites were located in the overlapping coding regions of the Rep and RepA genes, among which two positively selected sites were located at the N-terminus of the replicating proteins, while two and one positively selected sites were located at the C-terminus of RepA and Rep, respectively (Supplementary Table S8). Furthermore, no positively selected sites were detected in the CP and MP genes.

## Discussion

We conducted a large-scale systematic dynamics analysis of the global population of WDV by screening all complete gene sequences retrieved from GenBank. Our MCC trees show that WDV has two distinct evolutionary lineages, WDV-wheat and WDV-barley (Figure 1), which is consistent with the findings of previous reports (Lindsten and Vacke, 1991; Commandeur and Huth, 1999; Muhire et al., 2013; Schubert et al., 2014). Wheat is a hexaploid crop, while barley is a diploid crop. The differences between their genomes may lead to significant differences between barley and wheat in terms of resistance to WDV infection and proliferation in host cells. In response to different resistance mechanisms, WDV has host-specific host adaptations to better infect different hosts, resulting in the origin of different subspecies. Some reports showed that virions from WDV-barley and WDV-wheat strains do not infect barley and wheat plants (Abt et al., 2020), respectively, but other experiments showed that the virions of WDV-wheat could successfully infect barley plants (29), and the virions of WDV-barley could infect wheat plants (Lindsten and Vacke, 1991; Commandeur and Huth, 1999; Schubert et al., 2007; Kundu et al., 2009; Ramsell et al., 2009). According to the MCC trees that we reconstructed, some isolates of WDV-barley were derived from wheat, and some isolates of WDV-wheat has been derived from barley (Figure 1). Based on these results, we can confirm that there do not appear to be host differences between the wheat and barley strains of WDV, although the virus appears in two distinct evolutionary groups due to host-driven adaptation.

Furthermore, our results also showed that in WDV-barley, all isolates from Iran (except for one isolate in the CP dataset) were clustered together, and the rest were from Europe (Figures 1A,B). Similarly, in WDV-wheat, all isolates from Iran (except for one isolate in the CP dataset) still clustered on the same branch (Figure 1). There is increasing evidence that the genetic variability of many viruses is related to the geographical origin of virus isolates (Gao et al., 2017; Mao et al., 2019; Rao et al., 2020). Our results also show that the evolution of WDV was closely related to geographical location (Table 1; Supplementary Figures S1–S3). Geographic, environmental, or ecological isolation leads to genetic differentiation (Wang et al., 2013; MacDonald et al., 2020), and the emergence of environmental isolation and ecological isolation (IBE) comes from the spatially divergent adaptation of populations to different environmental or ecological conditions (Wang et al., 2013; Sánchez-Ramírez et al., 2018; MacDonald et al., 2020). Populations in environmental/ecological isolation necessarily imply differential selection in relation to abiotic or biological factors, which promoting genomic differentiation and limiting gene flow within species, thereby affecting the generation and maintenance of spatial population structure (Zhan and McDonald, 2013; MacDonald et al., 2022). The rapid accumulation of adaptive genetic variation in plant pathogen populations results from directional selection imposed by physical environments, pathogen biology and the ways of human intervention (Thrall et al., 2011). There are many similarities in the control of field plant diseases caused by viruses in different countries and regions. However, different farming methods and chemical pressures may lead to different adaptations of the virus. In addition, an interesting result is that the isolates from China clustered significantly together (Supplementary Figures S2–S4), even though the isolates are from different regions with different elevations and climatic conditions. This implies that physical factors such as the altitude and climatic experienced by the pathogens may not be causes of WDV population diversity.

WDV is transversely transmitted among host plants by *P. alienus*, and the geographical environment is one of the barriers to insect virus transmission. Although 25 million hectares of wheat and barley are grown in China every year (Ministry of Agriculture and Rural Affairs of China), no barley strains have been reported in the country. The most common form of geographic isolation is isolation by distance (IBD), which describes a pattern in which the genetic distance between individuals or populations is positively correlated with their geographic isolation (Wright, 1943). The extreme isolation by distance between China and Europe prevented *P. alienus* from transmitting WDV-barley into China. It is also important to note that although only wheat strains of WDV were distributed in China, some isolates were still collected from barley plants in the field. This further indicates that there is no difference in host plants between the wheat and barley strains of WDV. In addition, we did not observe adaptive evolution of WDV in other infected hosts, such as *A. sativa* (hexaploid) and *Secale cereale* (diploid). These results suggest that after the host-driven adaptation evolution of WDV, host-related evolution occurred again in response to the adaptation of its infected hosts.

Our results indicate that selection pressure plays an important role in the evolution of WDV, and the genes in WDV are subject to different degrees of purifying selection. The CP gene is subject to the strongest purifying selection, while MP gene is subjected to the weakest purification selection among the four genes of WDV. Positively selected sites were detected in only two genes encoding replication-related proteins (Supplementary Table S8). As the structural proteins of virions, coat proteins play a very important role in the life history of viruses. Meanwhile, for the viruses of *Geminiviridae*, the nuclear localization sequences (NLSs) of coat proteins are key to the entry of viral genetic material into the nucleus, and coat protein facilitates nuclear and cell-to-cell transport of the genome via interaction with the MP and NSP (Bennett and Agbandje-McKenna, 2020). Under high mutation rates, more important genes are bound to be subject to stronger purifying selection to prevent the effects of harmful mutations. Furthermore, an interesting result is that WDV-barley are subject to stronger purifying selection than WDV-wheat (Supplementary Table S7), and although only five positively selected sites were detected, they were all detected in a wheat strain of WDV (Figure 5; Supplementary Table S8). This may be related to the wider geographical distribution (China) of wheat strains, and the variation in host genetics, fungicide applications and agricultural practices among regions as means of natural selection led to the differential evolution between WDV-wheat and WDV-barley. Although the purifying selection of the Rep and RepA genes was less than that of the MP gene, all five positive selection sites were detected in the Rep and RepA genes. Both RepA and Rep have RNA silencing suppressor activities (Liu et al., 2014), and the mutability of specific sites may better resist the host plant’s defense mechanisms.

Because of their more stable genome structure, DNA viruses evolve at a slower rate than RNA viruses, which generally maintain a rate of approximately 10^−4^ substitutions/site/year (Pagan and Holmes, 2010; Fraile et al., 2011; Holmes, 2011). Unexpectedly, the evolutionary rate of WDV remained at 10^−4^ substitutions/site/year, and the results were similar to those of many RNA viruses. More surprisingly, the evolutionary rate of the MP gene was 1.309 × 10^−3^ substitutions/site/year (95% credibility interval: 7.117 × 10^−4^–1.993 × 10^−3^), which is even faster than that of some RNA viruses (Pagan and Holmes, 2010; Gao et al., 2020). Our results suggest that the evolutionary rate of WDV varies among genes, which is related to the function of the proteins encoded by the genes. Moreover, the TMRCAs of WDV obtained from the datasets with different coding regions were not the same, and the TMRCA of WDV obtained from the whole–genome coding region dataset was 1746 CE (95% credibility interval: 1517 CE–1893 CE). Furthermore, the TMRCA of each WDV gene obtained from the barley strain datasets was earlier than that from the wheat strain datasets, indicating that WDV may have first appeared in barley plants. In addition, the evolutionary rate of WDV-wheat was faster than that of WDV-barley (except for the mp gene, Supplementary Table S4). The difference in geographical distribution between wheat and barley strains of WDV may have contributed to this difference. This evolutionary difference between wheat and barley strains of WDV may be related to wheat strains having more complex plant–virus–environment relationships. Two host-driven adaptations of WDV resulted in distinct differentiation between WDV-wheat and WDV-barley. Moreover, only wheat strains were detected in China, which not only resulted in a larger population size of WDV-wheat than WDV-barley, but also made wheat strains face more complex environmental factors than barley strains.

Wheat and barley are the most important crops originating in the Fertile Crescent in Western Asia (Heun et al., 1997; Dubcovsky and Dvorak, 2007; Allaby et al., 2008; Brown et al., 2009; Pankin et al., 2018), and parts of Iran belong to the Fertile Crescent in Western Asia. As expected, our results identify that it is a high probability that WDV originated in Iran, and that the root posterior probabilities for Iran are much higher than that for China, Eastern Europe and Western Europe, which is supported by the whole–genome coding region and CP datasets (Figure 1; Supplementary Table S5). In addition, we obtained the same result by analysing the CP gene, which contains more isolates from Hungary (Figure 1B; Supplementary Table S5). Furthermore, our results, including the population history and TMRCA of WDV strains in barley and wheat, suggest that WDV may have originated in barley (Figure 3; Supplementary Table S4). The outbreak of WDV in the Fertile Crescent in Western Asia resulted in two distinct evolutionary lineages due to host adaptation. Our analysis of WDV migration pathways suggests that wheat strain of WDV spread from the Fertile Crescent in Western Asia to Eastern Europe before the 1840s, while WDV (both WDV-barley and -wheat) from the Fertile Crescent in Western Asia to Western Europe after 30 years. Surprisingly, Eastern European strain of WDV-barley only spread from Western Europe in the early 1960s. The wheat strain of WDV then spread to China in the early 1970s and from the middle reaches of the Yellow River to other major wheat–producing areas in China (Figure 2; Supplementary Table S5). Our other study (Wei et al., 2023) showed that the middle reaches of the Yellow River (Shaanxi and Shanxi provinces) are also a major emigration region of BYDV (barley yellow dwarf virus).

We performed a molecular epidemiology of WDV based on the whole–genome coding region sequences and unveiled the evolutionary history of the virus. During the evolutionary history of WDV, there were two episodes of adaptive evolution in its infected host plants. The first episode, which occurred in the Fertile Crescent in Western Asia, led to the emergence of two different evolutionary lineages of WDV, namely, wheat and barley strains, and the second episode allowed the two different strains to successfully infect hexaploid and diploid host plants. The TMRCA and BSP results indicate that WDV first appeared in barley plants. The population of WDV-barley began to expand in the early 1990s, and the population of the wheat strain began to expand in the early 2000s, while the population of the barley strain began to decline. After 2010, the populations of WDV-barley and WDV-wheat decreased to basically the same size as the population before expansion. Through pedigree and geographic analysis, we found that WDV probably originated in the Fertile Crescent in Western Asia and spread to other regions, and that Western Europe and the middle reaches of the Yellow River were the main export regions for WDV. While these results have provided new understanding into the evolutionary history of WDV, we may need variant information collected after 2017 for further assess the evolutionary history of WDV.

## Data availability statement

The original contributions presented in the study are included in the article/Supplementary material, further inquiries can be directed to the corresponding author.

## Author contributions

SW: Conceptualization, Data curation, Methodology, Software, Visualization, Writing – original draft, Writing – review & editing. LL: Visualization, Writing – review & editing. GC: Visualization, Writing – review & editing. HY: Conceptualization, Writing – review & editing. LH: Software, Writing – review & editing. GG: Conceptualization, Writing – review & editing. PL: Conceptualization, Methodology, Writing – review & editing. MZ: Conceptualization, Funding acquisition, Project administration, Writing – original draft, Writing – review & editing.
