# Supplementary figures and images for: Molecular evolution and phylogeographic analysis of wheat dwarf virus
Source: Front Microbiol. 2024 Feb 14;15:1314526. doi: 10.3389/fmicb.2024.1314526 (PMC10901289; doi:10.3389/fmicb.2024.1314526)

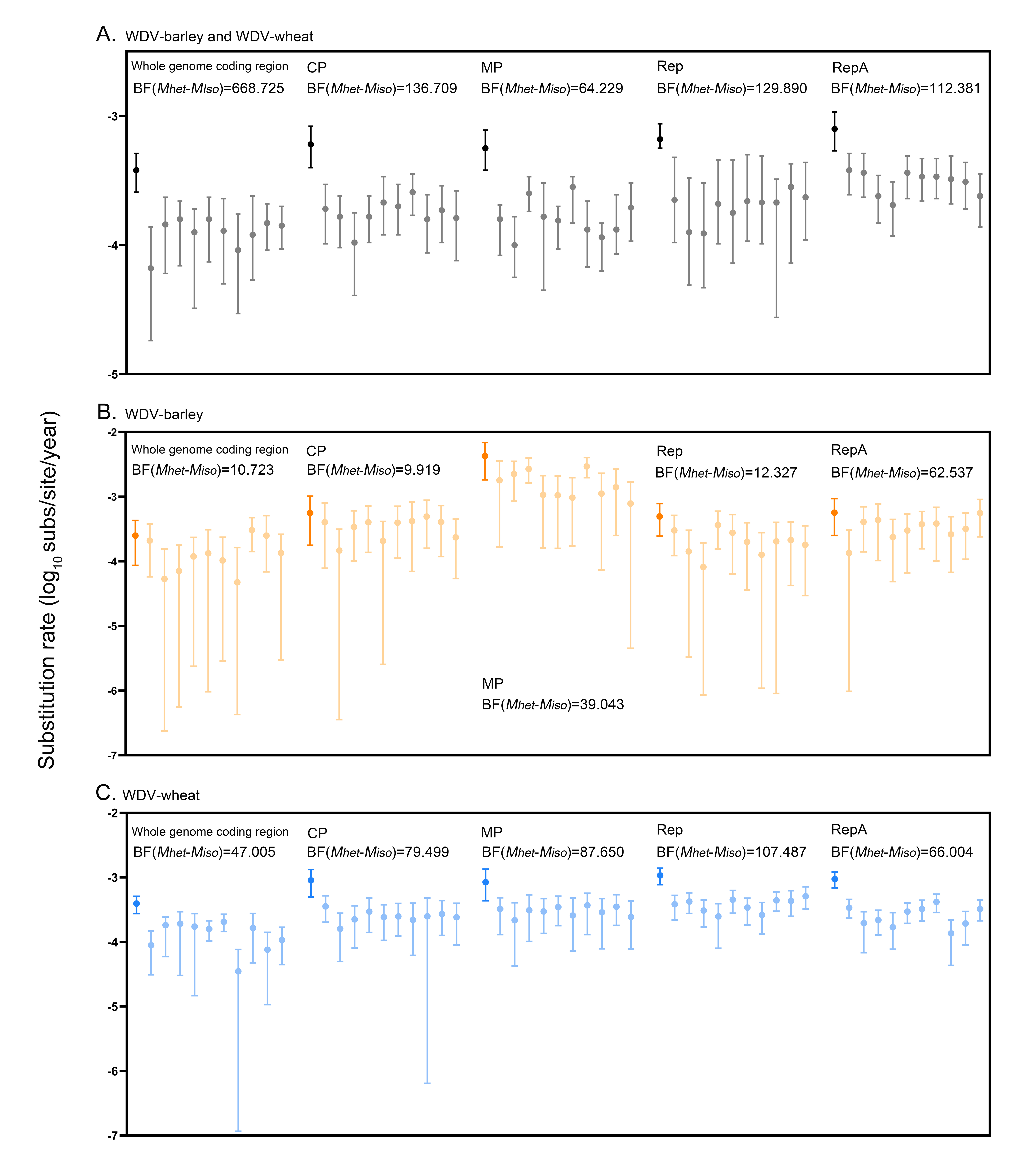

Supplement: Supplementary file 1 [file Image_1.JPEG]

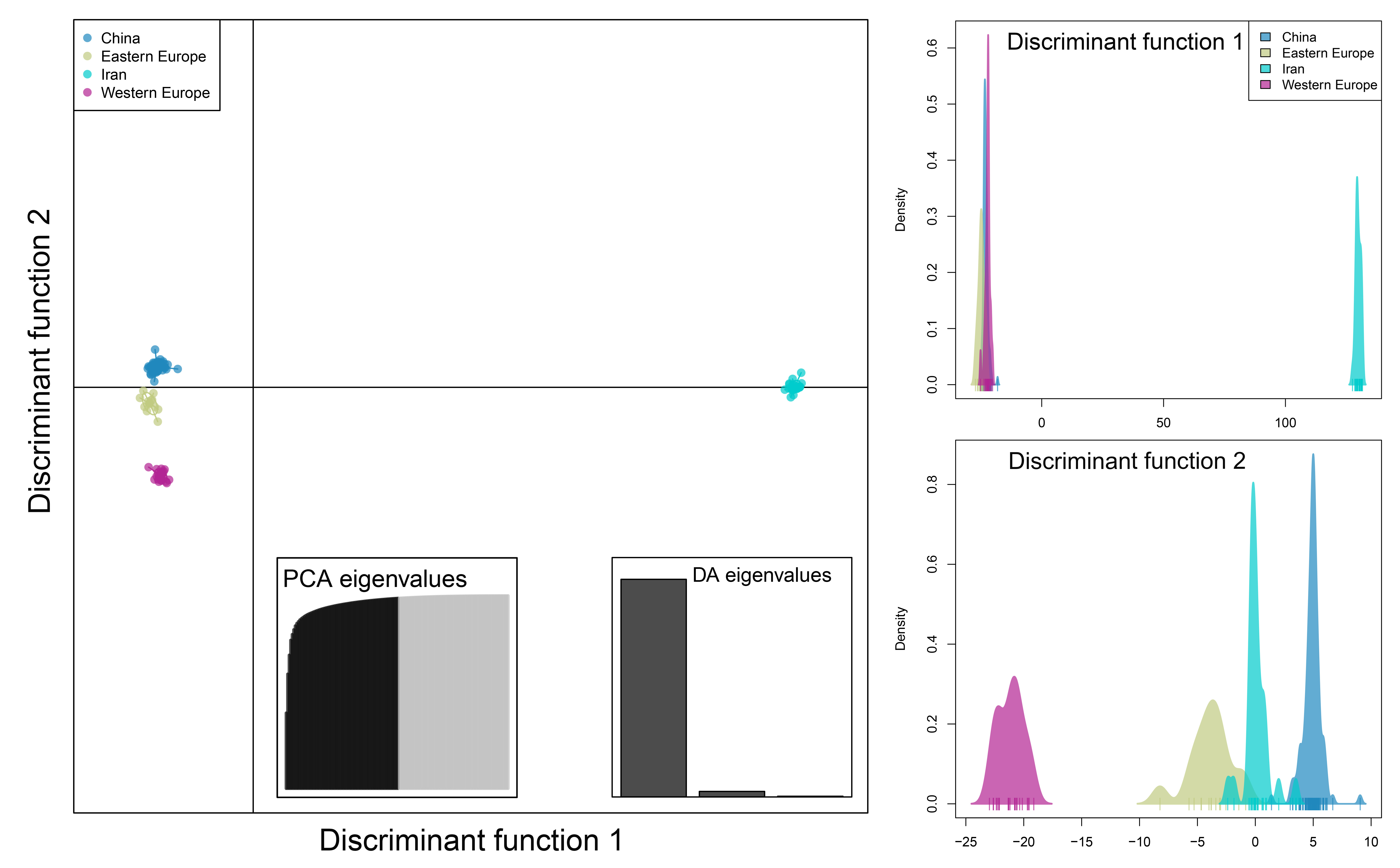

Supplement: Supplementary file 2 [file Image_2.JPEG]
