# Supplementary material for: Molecular evolution and phylogeographic analysis of wheat dwarf virus
Source: Front Microbiol. 2024 Feb 14;15:1314526. doi: 10.3389/fmicb.2024.1314526 (PMC10901289; doi:10.3389/fmicb.2024.1314526)
Supplement: Supplementary file 5 [file Table_1.docx]

Supplementary Table 1. Isolates of WDV used in this study.

| GenBank ID | Isolate | Collection date | *Host* | Genes |
| --- | --- | --- | --- | --- |
| Austria | | | | |
| HF968646 | EcoKl1 | May-2012 | *Hordeum vulgare* | MP, CP, Rep, RepA |
| HF968647 | EcoKl2 | May-2012 | *Hordeum vulgare* | MP, CP, Rep, RepA |
| HF968648 | EcoKl3 | May-2012 | *Hordeum vulgare* | MP, CP, Rep, RepA |
| HF968649 | EcoKl4 | May-2012 | *Hordeum vulgare* | MP, CP, Rep, RepA |
| Bulgaria | | | | |
| AM989927 | Bg17 | 2006 | *Hordeum vulgare* | MP, CP, Rep, RepA |
| China: Gansu | | | | |
| EF536859 | GSGG05-1 | Apr-2005 | *Triticum aestivum* | MP, CP, Rep, RepA |
| EF536860 | GSGG05-2 | Apr-2005 | *Triticum aestivum* | MP, CP, Rep, RepA |
| KJ536078 | GSGG07-1 | 15-Apr-2007 | *Triticum aestivum* | MP, CP, Rep, RepA |
| KJ536079 | GSGG07-3 | 15-Apr-2007 | *Triticum aestivum* | MP, CP, Rep, RepA |
| KJ536080 | GSTS07-1 | 16-Apr-2007 | *Triticum aestivum* | MP, CP, Rep, RepA |
| KJ536081 | GSTS07-2 | 16-Apr-2007 | *Triticum aestivum* | MP, CP, Rep, RepA |
| KJ536082 | GSTS07-4 | 16-Apr-2007 | *Triticum aestivum* | MP, CP, Rep, RepA |
| KJ536083 | GSTS07-5 | 16-Apr-2007 | *Triticum aestivum* | MP, CP, Rep, RepA |
| KJ536084 | GSTS07-7 | 16-Apr-2007 | *Triticum aestivum* | MP, CP, Rep, RepA |
| KJ536085 | GSTS07-8 | 16-Apr-2007 | *Triticum aestivum* | MP, CP, Rep, RepA |
| KJ536137 | GSGG09-5 | 15-Apr-2009 | *Triticum aestivum* | MP, CP, Rep, RepA |
| KJ536136 | GSGG09-8 | 15-Apr-2009 | *Triticum aestivum* | MP, CP, Rep, RepA |
| China: Guizhou | | | | |
| KJ536086 | GZGY07-2 | 17-Mar-2007 | *Triticum aestivum* | MP, CP, Rep, RepA |
| KJ536087 | GZGY07-3 | 17-Mar-2007 | *Triticum aestivum* | MP, CP, Rep, RepA |
| KJ536088 | GZGY07-5 | 17-Mar-2007 | *Triticum aestivum* | MP, CP, Rep, RepA |
| China: Hebei | | | | |
| EF536862 | HBSJZ04 | 04-2004 | *Triticum aestivum* | MP, CP, Rep, RepA |
| EF536863 | HBSJZ06-3 | 2006 | *Triticum aestivum* | MP, CP, Rep, RepA |
| EF536864 | HBSJZ06-4 | 2006 | *Triticum aestivum* | MP, CP, Rep, RepA |
| EF536865 | HBSJZ06-5 | 2006 | *Triticum aestivum* | MP, CP, Rep, RepA |
| EF536866 | HBSJZ06-6 | 2006 | *Triticum aestivum* | MP, CP, Rep, RepA |
| EF536867 | HBSJZ06-7 | 2006 | *Triticum aestivum* | MP, CP, Rep, RepA |
| EF536868 | HBSJZ06-9 | 2006 | *Triticum aestivum* | MP, CP, Rep, RepA |
| EF536869 | HBSJZ06-10 | 2006 | *Triticum aestivum* | MP, CP, Rep, RepA |
| EF536870 | HBSJZ06-11 | 2006 | *Triticum aestivum* | MP, CP, Rep, RepA |
| EF536871 | HBSJZ06-12 | 2006 | *Triticum aestivum* | MP, CP, Rep, RepA |
| JQ647470 | HBSJZ10-10 | 17-Apr-2010 | *Triticum aestivum* | CP, Rep, RepA |
| JQ647471 | HBSJZ10-17 | 17-Apr-2010 | *Triticum aestivum* | MP, CP, Rep, RepA |
| JQ647472 | HBSJZ10-18 | 17-Apr-2010 | *Triticum aestivum* | MP, CP, Rep, RepA |
| JQ647473 | HBSJZ10-20 | 17-Apr-2010 | *Triticum aestivum* | MP, CP, Rep, RepA |
| JQ647474 | HBSJZ10-21 | 17-Apr-2010 | *Triticum aestivum* | MP, CP, Rep, RepA |
| JQ647475 | HBSJZ10-22 | 17-Apr-2010 | *Triticum aestivum* | MP, CP, Rep, RepA |
| JQ647476 | HBSJZ10-23 | 17-Apr-2010 | *Triticum aestivum* | MP, CP, Rep, RepA |
| KJ536089 | HBSJZ07-1 | 18-Apr-2007 | *Triticum aestivum* | MP, CP, Rep, RepA |
| KJ536132 | HBSJZ08-1 | 17-Apr-2008 | *Triticum aestivum* | MP, CP, Rep, RepA |
| China: Henan | | | | |
| EF536861 | HNZZ05 | 2005 | *Triticum aestivum* | MP, CP, Rep, RepA |
| KJ536090 | HNJZ07-1 | 01-Apr-2007 | *Triticum aestivum* | MP, CP, Rep, RepA |
| KJ536091 | HNJZ07-2 | 01-Apr-2007 | *Triticum aestivum* | MP, CP, Rep, RepA |
| KJ536092 | HNZZ08-1 | 30-Mar-2008 | *Triticum aestivum* | MP, CP, Rep, RepA |
| KJ536093 | HNZZ08-4 | 30-Mar-2008 | *Triticum aestivum* | MP, CP, Rep, RepA |
| KJ536094 | HNZZ08-6 | 30-Mar-2008 | *Triticum aestivum* | MP, CP, Rep, RepA |
| KJ536095 | HNZZ08-7 | 30-Mar-2008 | *Triticum aestivum* | MP, CP, Rep, RepA |
| KJ536127 | HNZD08-3 | 01-Apr-2008 | *Triticum aestivum* | MP, CP, Rep, RepA |
| KJ536128 | HNHYK08-4 | 02-Apr-2008 | *Triticum aestivum* | MP, CP, Rep, RepA |
| China: Hubei | | | | |
| KJ536126 | HBWH07-2 | 15-Mar-2007 | *Avena sativa* | MP, CP, Rep, RepA |
| China: Ningxia | | | | |
| KJ536096 | NXYC07-14 | 20-Apr-2007 | *Triticum aestivum* | MP, CP, Rep, RepA |
| KJ536097 | NXYC07-16 | 20-Apr-2007 | *Triticum aestivum* | MP, CP, Rep, RepA |
| KJ536098 | NXYC07-3 | 20-Apr-2007 | *Triticum aestivum* | MP, CP, Rep, RepA |
| KJ536099 | NXYC07-5 | 20-Apr-2007 | *Triticum aestivum* | MP, CP, Rep, RepA |
| KJ536100 | NXYN07-2 | 20-Apr-2007 | *Triticum aestivum* | MP, CP, Rep, RepA |
| KJ536101 | NXYN07-3 | 20-Apr-2007 | *Triticum aestivum* | MP, CP, Rep, RepA |
| China: Qinghai | | | | |
| JQ647477 | QHLD10-1 | 15-Jun-2010 | *Triticum aestivum* | MP, CP, Rep, RepA |
| JQ647478 | QHLD10-8 | 15-Jun-2010 | *Triticum aestivum* | MP, CP, Rep, RepA |
| JQ647479 | QHLD10-9 | 15-Jun-2010 | *Triticum aestivum* | MP, CP, Rep, RepA |
| JQ647480 | QHLD10-15 | 15-Jun-2010 | *Triticum aestivum* | MP, CP, Rep, RepA |
| JQ647481 | QHLD10-18 | 15-Jun-2010 | *Triticum aestivum* | MP, CP, Rep, RepA |
| JQ647482 | QHLD10-19 | 15-Jun-2010 | *Triticum aestivum* | MP, CP, Rep, RepA |
| KJ536147 | QHXN08-29 | 15-Jun-2008 | Hullessbarley | MP, CP, Rep, RepA |
| KJ536148 | QHXN08-26 | 15-Jun-2008 | Hullessbarley | MP, CP, Rep, RepA |
| KJ536149 | QHXN08-17 | 15-Jun-2008 | Hullessbarley | MP, CP, Rep, RepA |
| KJ536150 | QHXN08-11 | 15-Jun-2008 | Hullessbarley | MP, CP, Rep, RepA |
| China: Shaanxi | | | | |
| EF536877 | SXYL05-1 | 2005 | *Triticum aestivum* | MP, CP, Rep, RepA |
| EF536878 | SXYL05-2 | 2005 | *Triticum aestivum* | MP, CP, Rep, RepA |
| EF536879 | SXYL05-3 | 2005 | *Triticum aestivum* | MP, CP, Rep, RepA |
| EF536880 | SXYL05-4 | 2005 | *Triticum aestivum* | MP, CP, Rep, RepA |
| EF536881 | SXYL05-5 | 2005 | *Triticum aestivum* | MP, CP, Rep, RepA |
| EF536882 | SXYL05-6 | 2005 | *Triticum aestivum* | MP, CP, Rep, RepA |
| JQ647488 | SXHC10-5 | 12-May-2010 | *Triticum aestivum* | MP, CP, Rep, RepA |
| JQ647489 | SXHC10-6 | 12-May-2010 | *Triticum aestivum* | MP, CP, Rep, RepA |
| JQ647490 | SXHC10-7 | 12-May-2010 | *Triticum aestivum* | MP, CP, Rep, RepA |
| JQ647491 | SXHC10-8 | 12-May-2010 | *Triticum aestivum* | MP, CP, Rep, RepA |
| JQ647492 | SXHC10-9 | 12-May-2010 | *Triticum aestivum* | MP, CP, Rep, RepA |
| JQ647493 | SXHC10-10 | 12-May-2010 | *Triticum aestivum* | MP, CP, Rep, RepA |
| JQ647494 | SXHC10-11 | 12-May-2010 | *Triticum aestivum* | MP, CP, Rep, RepA |
| JQ647495 | SXHC10-12 | 12-May-2010 | *Triticum aestivum* | MP, CP, Rep, RepA |
| JQ647496 | SXHC10-14 | 12-May-2010 | *Triticum aestivum* | MP, CP, Rep, RepA |
| JQ647497 | SXHC10-15 | 12-May-2010 | *Triticum aestivum* | MP, CP, Rep, RepA |
| JQ647498 | SXHC10-16 | 12-May-2010 | *Triticum aestivum* | MP, CP, Rep, RepA |
| JQ647499 | SXHC10-17 | 12-May-2010 | *Triticum aestivum* | MP, CP, Rep, RepA |
| JQ647500 | SXHC10-18 | 12-May-2010 | *Triticum aestivum* | MP, CP, Rep, RepA |
| KJ536108 | SXHC07-10 | 15-May-2007 | *Triticum aestivum* | MP, CP, Rep, RepA |
| KJ536109 | SXHC07-14 | 15-May-2007 | *Triticum aestivum* | MP, CP, Rep, RepA |
| KJ536110 | SXHC07-19 | 15-May-2007 | *Triticum aestivum* | MP, CP, Rep, RepA |
| KJ536111 | SXHC07-2 | 15-May-2007 | *Triticum aestivum* | MP, CP, Rep, RepA |
| KJ536112 | SXHC07-28 | 15-May-2007 | *Triticum aestivum* | MP, CP, Rep, RepA |
| KJ536113 | SXHC07-29 | 15-May-2007 | *Triticum aestivum* | MP, CP, Rep, RepA |
| KJ536114 | SXHC07-3 | 15-May-2007 | *Triticum aestivum* | MP, CP, Rep, RepA |
| KJ536115 | SXHC07-31 | 15-May-2007 | *Triticum aestivum* | MP, CP, Rep, RepA |
| KJ536121 | SXYL07-2 | 17-Apr-2007 | *Triticum aestivum* | MP, CP, Rep, RepA |
| KJ536122 | SXYL07-4 | 17-Apr-2007 | *Triticum aestivum* | MP, CP, Rep, RepA |
| KJ536123 | SXYL07-5 | 17-Apr-2007 | *Triticum aestivum* | MP, CP, Rep, RepA |
| KJ536124 | SXYL07-8 | 17-Apr-2007 | *Triticum aestivum* | MP, CP, Rep, RepA |
| KJ536138 | SXHC09-1 | 13-May-2009 | *Triticum aestivum* | MP, CP, Rep, RepA |
| KJ536139 | SXHC09-11 | 13-May-2009 | *Triticum aestivum* | MP, CP, RepA |
| KJ536140 | SXHC09-27 | 13-May-2009 | *Triticum aestivum* | MP, CP |
| China: Shandong | | | | |
| KJ536102 | SDJN07-2 | 10-May-2007 | *Triticum aestivum* | MP, CP, Rep, RepA |
| KJ536103 | SDJN07-3 | 10-May-2007 | *Triticum aestivum* | MP, CP, Rep, RepA |
| KJ536104 | SDJN07-8 | 10-May-2007 | *Triticum aestivum* | MP, CP, Rep, RepA |
| KJ536105 | SDJN07-5 | 10-May-2007 | *Triticum aestivum* | MP, CP, Rep, RepA |
| China: Shanxi | | | | |
| EF536872 | SXTY05-1 | 2005 | *Triticum aestivum* | MP, CP, Rep, RepA |
| EF536873 | SXTY05-2 | 2005 | *Triticum aestivum* | MP, CP, Rep, RepA |
| EF536874 | SXTY05-3 | 2005 | *Triticum aestivum* | MP, CP, Rep, RepA |
| EF536875 | SXYC05-2 | 2005 | *Triticum aestivum* | MP, CP, Rep, RepA |
| EF536876 | SXYC05-3 | 2005 | *Triticum aestivum* | MP, CP, Rep, RepA |
| JQ647486 | SXDT10-2 | 08-May-2010 | *Triticum aestivum* | MP, CP, Rep, RepA |
| JQ647487 | SXDT10-3 | 08-May-2010 | *Triticum aestivum* | MP, CP, Rep, RepA |
| JQ647501 | SXTY10-9 | 08-May-2010 | *Triticum aestivum* | MP, CP, Rep, RepA |
| JQ647502 | SXTY10-18 | 08-May-2010 | *Triticum aestivum* | MP, CP, Rep, RepA |
| JQ647503 | SXTY10-32 | 08-May-2010 | *Triticum aestivum* | MP, CP, Rep, RepA |
| JQ647504 | SXTY10-33 | 08-May-2010 | *Triticum aestivum* | MP, CP, Rep, RepA |
| JQ647505 | SXTY10-35 | 08-May-2010 | *Triticum aestivum* | MP, CP, Rep, RepA |
| JQ647506 | SXTY10-39 | 08-May-2010 | *Triticum aestivum* | MP, CP, Rep, RepA |
| JQ647507 | SXTY10-40 | 08-May-2010 | *Triticum aestivum* | MP, CP, Rep, RepA |
| KJ536106 | SXDT07-2 | 15-Jun-2007 | *Triticum aestivum* | MP, CP, Rep, RepA |
| KJ536107 | SXDT07-3 | 15-Jun-2007 | *Triticum aestivum* | MP, CP, Rep, RepA |
| KJ536116 | SXLF07-1 | 27-Apr-2007 | *Triticum aestivum* | MP, CP, Rep, RepA |
| KJ536117 | SXLF07-2 | 27-Apr-2007 | *Triticum aestivum* | MP, CP, Rep, RepA |
| KJ536118 | SXLF07-5 | 27-Apr-2007 | *Triticum aestivum* | MP, CP, Rep, RepA |
| KJ536119 | SXTY07-3 | 08-May-2007 | *Triticum aestivum* | MP, CP, Rep, RepA |
| KJ536120 | SXTY07-6 | 08-May-2007 | *Triticum aestivum* | MP, CP, Rep, RepA |
| China: Sichuan | | | | |
| JQ647483 | SCCD10-2 | 12-Mar-2010 | *Triticum aestivum* | MP, CP, Rep, RepA |
| JQ647484 | SCCD10-3 | 12-Mar-2010 | *Triticum aestivum* | MP, CP, Rep, RepA |
| JQ647485 | SCCD10-6 | 12-Mar-2010 | *Triticum aestivum* | MP, CP, Rep, RepA |
| KJ536125 | SCCD07-1 | 12-Mar-2007 | *Triticum aestivum* | MP, CP, Rep, RepA |
| China: Xinjiang | | | | |
| KJ536133 | XJWLMQ08-3 | 15-May-2008 | *Triticum aestivum* | MP, CP, Rep, RepA |
| KJ536134 | XJWLMQ08-5 | 15-May-2008 | *Triticum aestivum* | MP, CP, Rep, RepA |
| KJ536135 | XJWLMQ08-12 | 15-May-2008 | *Triticum aestivum* | MP, CP, Rep, RepA |
| China: Tibet | | | | |
| JQ647508 | TIBET10-2 | 25-Jun-2010 | *Triticum aestivum* | CP, Rep, RepA |
| China: Yunnan | | | | |
| EF536883 | YNKM06-1 | 2006 | *Triticum aestivum* | MP, CP, Rep, RepA |
| EF536884 | YNKM06-3 | 2006 | *Triticum aestivum* | MP, CP, Rep, RepA |
| EF536885 | YNKM06-4 | 2006 | *Triticum aestivum* | MP, CP, Rep, RepA |
| EF536886 | YNKM06-2 | 2006 | *Triticum aestivum* | MP, CP, Rep, RepA |
| EU541489 | YNKM07-29 | 2005 | *Triticum aestivum* | MP, CP, Rep, RepA |
| KJ536129 | YNKM08-10 | 15-Mar-2008 | *Triticum aestivum* | MP, CP, Rep, RepA |
| KJ536130 | YNKM08-2 | 15-Mar-2008 | *Triticum aestivum* | MP, CP, Rep, RepA |
| KJ536131 | YNKM08-8 | 15-Mar-2008 | *Triticum aestivum* | MP, CP, Rep, RepA |
| KJ536141 | YNKM08-13 | 15-Mar-2008 | *Hordeum vulgare* | MP, CP, Rep, RepA |
| KJ536142 | YNKM07-31 | 15-Mar-2008 | *Hordeum vulgare* | MP, CP, Rep, RepA |
| KJ536143 | YNKM07-35 | 15-Mar-2008 | *Hordeum vulgare* | MP, CP, Rep, RepA |
| KJ536144 | YNKM07-38 | 15-Mar-2008 | *Hordeum vulgare* | MP, CP, Rep, RepA |
| KJ536145 | YNKM07-39 | 15-Mar-2008 | *Hordeum vulgare* | MP, CP, Rep, RepA |
| KJ536146 | YNKM07-43 | 15-Mar-2008 | *Hordeum vulgare* | MP, CP, Rep, RepA |
| Czech Republic | | | | |
| AM296019 | Cz19 | 2001 | *Hordeum vulgare* | CP, RepA |
| Estonia | | | | |
| MK193742 |  | 2017-07-17 | *Triticum aestivum* | MP, Rep, RepA |
| France | | | | |
| HF968637 | FR68WDV_ KpnKl1 | May-2010 | *Triticum aestivum* | MP, CP, Rep, RepA |
| HF968638 | FR68WDV_ KpnKl2 | May-2010 | *Triticum aestivum* | MP, CP, Rep, RepA |
| MN594280 | b1 | 2011 | *Hordeum vulgare* | MP, CP, Rep, RepA |
| MN594281 | w1 | 2011 | *Triticum aestivum* | MP, CP, Rep, RepA |
| Germany | | | | |
| AM296018 | SxA18 | 2003 | *Avena sativa* | MP, CP, Rep, RepA |
| AM296020 | McP20 | 2001 | *Hordeum vulgare* | MP, CP, Rep, RepA |
| AM296021 | BB21 | 2004 | *Secale cereale* | MP, CP, Rep, RepA |
| AM296022 | SxA22 | 2002 | *Lolium perenne* | MP, CP, Rep, RepA |
| AM296023 | SxA23 | 2004 | *Triticum aestivum* | MP, CP, Rep, RepA |
| AM296024 | Sx24 | 2002 | *Triticum aestivum* | MP, CP, Rep, RepA |
| AM411651 | Baden-Wuerttemberg 1 | 2005 | *Hordeum vulgare* | MP, CP, Rep, RepA |
| AM411652 | Baden-Wuerttemberg 2 | 2005 | *Hordeum vulgare* | MP, CP, Rep, RepA |
| AM921649 | SABgl2-11 | 12-Mar-2007 | *Hordeum vulgare* | MP, CP, Rep, RepA |
| AM921991 | SA41WhEcoFL6 | 12-Mar-2007 | *Triticum aestivum* | MP, CP, Rep, RepA |
| AM921992 | SA1EcoFL2 | 12-Mar-2007 | *Hordeum vulgare* | MP, CP, Rep, RepA |
| AM921993 | SA12PstFL1 | 12-Mar-2007 | *Hordeum vulgare* | MP, CP, Rep, RepA |
| AM921994 | SA6PstFL1 | 12-Mar-2007 | *Hordeum vulgare* | MP, CP, Rep, RepA |
| AM921995 | SA45Ecogr16 | 12-Mar-2007 | *Hordeum vulgare* | MP, CP |
| AM921996 | SA45Ecogr11 | 12-Mar-2007 | *Hordeum vulgare* | MP, CP |
| AM922260 | SA45EcoFL38 | 12-Mar-2007 | *Hordeum vulgare* | MP, CP, Rep, RepA |
| AM922261 | SA43EcoFL23 | 12-Mar-2007 | *Hordeum vulgare* | MP, CP, Rep, RepA |
| AM922262 | SA12EcoFL2 | 12-Mar-2007 | *Hordeum vulgare* | MP, CP, Rep, RepA |
| AM922263 | SA43Ecogr15 | 12-Mar-2007 | *Hordeum vulgare* | Rep, RepA |
| AM942044 | SxA 57 | 31-Mar-2007 | *Triticum aestivum* | MP, CP, Rep, RepA |
| AM942045 | SxA 36 | 31-Mar-2007 | *Triticum aestivum* | MP, CP, Rep, RepA |
| AM980882 |  | 31-Mar-2007 | *Hordeum vulgare* | MP, CP |
| AM980883 | SxA43-16 | 31-Mar-2007 | *Hordeum vulgare* | MP, Rep, RepA |
| HG422310 | Leutewitz_224 | May-2013 | *Triticum aestivum* | MP, CP, Rep, RepA |
| HG422311 | Leutewitz_30 | May-2013 | *Triticum aestivum* | MP, CP, Rep, RepA |
| HG422312 | Leutewitz_2 | May-2013 | *Hordeum vulgare* | MP, CP, Rep, RepA |
| HG422313 | Leutewitz_3 | May-2013 | *Hordeum vulgare* | MP, CP, Rep, RepA |
| HG422314 | Aschersleben 3 | Mar-2000 | *Hordeum vulgare* | MP, CP, Rep, RepA |
| HG422315 | Krostitz 1 | Mar-2001 | *Hordeum vulgare* | MP, CP, Rep, RepA |
| HG422316 | Aschersleben 1 | Sep-2001 | *Lolium perenne* | MP, CP, Rep, RepA |
| HG422317 | Alsleben 3 | Mar-2001 | *Triticum aestivum* | MP, CP, Rep, RepA |
| HG422318 | Alsleben 9 | Mar-2001 | *Triticum aestivum* | MP, CP, Rep, RepA |
| HF968650 | EcoKl38 | May-2009 | *Hordeum vulgare* | MP, CP, Rep, RepA |
| KJ473695 | Spelt8 | May-2008 | *Triticum aestivum* | MP, CP, Rep, RepA |
| KJ473696 | Spelt9 | May-2008 | *Triticum aestivum* | MP, CP, Rep, RepA |
| KJ473697 | Winter_rye_49 | May-2008 | *Secale cereale* | MP, CP, Rep, RepA |
| KJ473698 | Winter_rye_101 | May-2008 | *Secale cereale* | MP, CP, Rep, RepA |
| KJ473699 | Winter_rye_102 | May-2008 | *Secale cereale* | MP, CP, Rep, RepA |
| KJ473700 | Winter_Triticum aestivum_110 | May-2008 | *Triticum aestivum* | MP, CP, Rep, RepA |
| KJ473701 | Winter_Triticum aestivum_111 | May-2008 | *Triticum aestivum* | MP, CP, Rep, RepA |
| KJ473702 | Triticale_117 | May-2008 | triticale | MP, CP, Rep, RepA |
| KJ473703 | Triticale_118 | May-2008 | triticale | MP, CP, Rep, RepA |
| KJ473704 | Winter_Triticum aestivum_120 | May-2008 | *Triticum aestivum* | MP, CP, Rep, RepA |
| KJ473705 | Winter_Triticum aestivum_121 | May-2008 | *Triticum aestivum* | MP, CP, Rep, RepA |
| KJ473706 | Rye4_6 | May-2008 | *Secale cereale* | MP, CP, Rep, RepA |
| KJ473707 | Rye9_3 | May-2008 | *Secale cereale* | MP, CP, Rep, RepA |
| Hungary | | | | |
| AM040732 | WDV-B | 2005 | *Triticum aestivum* | MP, CP, Rep, RepA |
| AM040733 | WDV-F | 2005 | *Triticum aestivum* | MP, CP, Rep, RepA |
| AM747816 | Hordeum vulgare | 1999 | *Hordeum vulgare* | MP, CP, Rep, RepA |
| FM210034 | H07 | 2003 | *Hordeum vulgare* | MP, CP, Rep, RepA |
| FM999832 | D01 | Sep-2007 | *Hordeum vulgare* | MP, CP, Rep, RepA |
| FM999833 | HE | Sep-2007 | *Hordeum vulgare* | MP, CP, Rep, RepA |
| FN806785 | WDV-HU-2Marton | 2008 | *Triticum aestivum* | MP, CP, Rep, RepA |
| FN806786 | WDV-HU-Pula | 2008 | *Triticum aestivum* | MP, CP, Rep, RepA |
| JQ647454 | HUNGARY-PC10-1 | 29-Apr-2010 | *Triticum aestivum* | MP, CP, Rep, RepA |
| JQ647455 | HUNGARY-KP10-1 | 28-Apr-2010 | *Triticum aestivum* | CP, Rep, RepA |
| JQ647456 | HUNGARY-KP10-3 | 28-Apr-2010 | *Triticum aestivum* | MP, CP, Rep, RepA |
| JQ647457 | HUNGARY-KP10-4 | 28-Apr-2010 | *Triticum aestivum* | CP, Rep, RepA |
| JQ647458 | HUNGARY-KP10-5 | 28-Apr-2010 | *Triticum aestivum* | MP, CP, Rep, RepA |
| JQ647459 | HUNGARY-KP10-6 | 28-Apr-2010 | *Triticum aestivum* | CP, Rep, RepA |
| JQ647460 | HUNGARY-KP10-8 | 28-Apr-2010 | *Triticum aestivum* | MP, CP, Rep, RepA |
| JQ647461 | HUNGARY-KP10-9 | 28-Apr-2010 | *Triticum aestivum* | CP, Rep, RepA |
| JQ647462 | HUNGARY-KP10-10 | 28-Apr-2010 | *Triticum aestivum* | CP, Rep, RepA |
| JQ647463 | HUNGARY-KP10-11 | 28-Apr-2010 | *Triticum aestivum* | CP, Rep, RepA |
| JQ647464 | HUNGARY-KP10-13 | 28-Apr-2010 | *Triticum aestivum* | CP, Rep, RepA |
| JQ647465 | HUNGARY-KP10-15 | 28-Apr-2010 | *Triticum aestivum* | CP, Rep, RepA |
| JQ647466 | HUNGARY-KP10-16 | 30-Apr-2010 | *Triticum aestivum* | MP, CP, Rep, RepA |
| JQ647467 | HUNGARY-MO10-1 | 30-Apr-2010 | *Triticum aestivum* | MP, CP, Rep, RepA |
| JQ647468 | HUNGARY-MO10-2 | 30-Apr-2010 | *Triticum aestivum* | MP, CP, Rep, RepA |
| JQ647469 | HUNGARY-MO10-3 | 30-Apr-2010 | *Triticum aestivum* | MP, CP, Rep, RepA |
| Iran | | | | |
| FJ620684 |  | May-2006 | *Hordeum vulgare* | MP, CP, Rep, RepA |
| JN791095 | Shahrekord | 03-Feb-2010 | *Triticum aestivum* | MP, CP |
| JN791096 | Bavanat | 03-Feb-2010 | *Hordeum vulgare* | MP, CP |
| KT958235 | SYZSH03 | 25-May-2015 | *Sorghum halepense* | MP, CP, Rep, RepA |
| KT958236 | SYZHS06 | 25-May-2015 | *Hordeum spontaneum* | MP, CP, Rep, RepA |
| KT958237 | SYZAF12 | 25-May-2015 | *Avena fatua* | MP, CP, Rep, RepA |
| KT958238 | SYZBC14 | 25-May-2015 | *Bromus commutatus* | MP, CP, Rep, RepA |
| KT958239 | IFNHV22 | 18-Sep-2009 | *Hordeum vulgare* | MP, CP, Rep, RepA |
| KT958240 | SYZEP26 | 25-May-2015 | *Eremopoa persica* | MP, CP, Rep, RepA |
| KT958241 | SYZHM32 | 25-May-2015 | *Hordeum murinum* | MP, CP, Rep, RepA |
| KT958242 | SYZLP33 | 25-May-2015 | *Lolium persicum* | MP, CP, Rep, RepA |
| KT958243 | SYZCD35 | 25-May-2015 | *Cynodon dactylon* | MP, CP, Rep, RepA |
| KT958244 | SYZAK54 | 25-May-2015 | *Aegilops kotschyi* | MP, CP, Rep, RepA |
| KT958245 | MRTTA56 | 30-May-2015 | *Triticum aestivum* | MP, CP, Rep, RepA |
| KT958246 | MRTTA59 | 30-May-2015 | *Triticum aestivum* | MP, CP, Rep, RepA |
| KT958247 | MRTTA60 | 30-May-2015 | *Triticum aestivum* | MP, CP, Rep, RepA |
| KT958248 | MRTTA64 | 30-May-2015 | *Triticum aestivum* | MP, CP, Rep, RepA |
| KT958249 | MRTTA66 | 30-May-2015 | *Triticum aestivum* | MP, CP, Rep, RepA |
| KT958250 | MRTTA71 | 30-May-2015 | *Triticum aestivum* | MP, CP, Rep, RepA |
| KT958251 | KLTHV03 | 10-Aug-2009 | *Hordeum vulgare* | MP, CP, Rep, RepA |
| KT958252 | RDLTA09 | 16-Aug-2009 | *Triticum aestivum* | MP, CP, Rep, RepA |
| KT958253 | MRTTA10 | 03-Oct-2009 | *Triticum aestivum* | MP, CP, Rep, RepA |
| KU877913 | SYZTA01-4 | 31-May-2015 | *Triticum aestivum* | MP, CP, Rep, RepA |
| KU877914 | SYZTA01-6 | 31-May-2015 | *Triticum aestivum* | MP |
| KU877915 | SYZTA03-NSI | 31-May-2015 | *Triticum aestivum* | MP, CP, Rep, RepA |
| KU877916 | IFNHV22-RCA | 14-Sep-2009 | *Hordeum vulgare* | MP, CP, Rep, RepA |
| KU877917 | CRNTA01-1 | 15-Sep-2009 | *Triticum aestivum* | MP, CP, Rep, RepA |
| KU877918 | CRNTA01 | 15-Sep-2009 | *Triticum aestivum* | MP, CP, Rep, RepA |
| KU877919 | SVHTA02-10 | 22-Sep-2009 | *Triticum aestivum* | MP |
| KU877920 | SVHTA02 | 22-Sep-2009 | *Triticum aestivum* | MP, CP, Rep, RepA |
| KU877921 | VRKHV04 | 18-Sep-2009 | *Hordeum vulgare* | MP, CP, Rep, RepA |
| KU877922 | YESHV07 | 20-Sep-2009 | *Hordeum vulgare* | MP, CP, Rep, RepA |
| KU877923 | IILTA13 | 24-Sep-2009 | *Triticum aestivum* | MP, CP, Rep, RepA |
| KU877924 | KRJTA16 | 30-Sep-2009 | *Triticum aestivum* | MP, CP, Rep, RepA |
| KU877925 | FRDTA17 | 23-Sep-2009 | *Triticum aestivum* | MP, CP, Rep, RepA |
| KU877926 | KHDTA2-RCA | 27-Sep-2009 | *Triticum aestivum* | MP, CP, Rep, RepA |
| KU877927 | TBZTA28 | 17-Sep-2009 | *Triticum aestivum* | MP, CP, Rep, RepA |
| KU877928 | KSHTA30 | 29-Sep-2009 | *Triticum aestivum* | MP, CP, Rep, RepA |
| KU933915 | SYZTA01-2 | 31-May-2015 | *Triticum aestivum* | MP |
| KU933916 | SVHTA02-15 | 22-Sep-2009 | *Triticum aestivum* | MP, CP, Rep, RepA |
| KU933917 | AZDHV09-1 | 18-Sep-2009 | *Hordeum vulgare* | MP, CP, Rep, RepA |
| KU933918 | KERHV11-1 | 10-Sep-2009 | *Hordeum vulgare* | MP, CP, Rep, RepA |
| KU933919 | KERHV11-RCA | 10-Sep-2009 | *Hordeum vulgare* | MP, CP, Rep, RepA |
| KU933920 | TEHTA19 | 13-Sep-2009 | *Triticum aestivum* | MP, CP, Rep, RepA |
| KX212082 | BJHTA01 | 01-May-2015 | *Triticum aestivum* | MP, CP, Rep, RepA |
| KX212083 | BJHHV01 | 01-May-2015 | *Hordeum vulgare* | MP, CP, Rep, RepA |
| KX533461 | WDV_IR_CZ2_2013 | 2013 | *Psammotettix alienus* | MP, CP, Rep, RepA |
| KX533462 | WDV_IR_CZ3_2013 | 2013 | *Psammotettix alienus* | MP, CP, Rep, RepA |
| KX533463 | WDV_IR_CZ4_2013 | 2013 | *Psammotettix alienus* | MP, CP, Rep, RepA |
| KX533464 | WDV_IR_CZ5_2013 | 2013 | *Psammotettix alienus* | MP, CP, Rep, RepA |
| KX533465 | WDV_IR_CZ7_2013 | 2013 | *Psammotettix alienus* | MP, CP, Rep, RepA |
| KX889118 | IRI-B | 01-May-2015 | *Hordeum vulgare* | MP, CP, Rep, RepA |
| KY679016 | MRDTA22-1 | 30-Jun-2016 | *Triticum aestivum* | MP, CP, Rep, RepA |
| KY679017 | MRDHV02-4 | 30-Jun-2016 | *Hordeum vulgare* | MP, CP, Rep, RepA |
| KY793552 | MRDTA24-1 | 30-Jun-2016 | *Triticum aestivum* | MP |
| KY793553 | MRDHV04-2 | 30-Jun-2016 | *Psammotettix alienus* | MP |
| MH477616 | ADAHV_1 | 31-May-2017 | *Hordeum vulgare* | MP, CP, Rep, RepA |
| MH477617 | ADAHV_2 | 22-Sep-2017 | *Hordeum vulgare* | MP, CP, Rep, RepA |
| MH477618 | ADAHV_3 | 18-Sep-2017 | *Hordeum vulgare* | MP, CP, Rep, RepA |
| MH477619 | ADAHV_4 | 10-Sep-2017 | *Hordeum vulgare* | MP, CP, Rep, RepA |
| MH477620 | ADATA_1 | 10-Sep-2017 | *Triticum aestivum* | MP, CP, Rep, RepA |
| MH477621 | ADATA_2 | 13-Sep-2017 | *Triticum aestivum* | MP, CP, Rep, RepA |
| MH477622 | ADATA_3 | 13-Sep-2017 | *Triticum aestivum* | MP, CP, Rep, RepA |
| MH477623 | ADATA_4 | 13-Sep-2017 | *Triticum aestivum* | MP, CP, Rep, RepA |
| MH513492 | ADAHV_2_DF | 31-May-2017 | *Hordeum vulgare* | MP |
| MN688644 | SH1-HB | 21-Apr-2016 | *Hordeum vulgare* | MP, CP, Rep, RepA |
| Poland | | | | |
| KM079154 | Pol-WDV-W | May-2007 | *Hordeum vulgare* | MP, CP, Rep, RepA |
| KM079155 | WDV-B | May-2007 | *Hordeum vulgare* | MP, CP, Rep, RepA |
| KY781933 | WDV-Ant | Apr-2014 | *Triticum aestivum* | CP |
| KY781934 | WDV-Bol | Apr-2015 | *Hordeum vulgare* | CP |
| KY781935 | WDV-Dl | May-2016 | *Hordeum vulgare* | CP |
| KY781936 | WDV-Gl | Apr-2015 | *Triticum aestivum* | CP |
| KY781937 | WDV-Knd1 | May-2013 | *Triticum aestivum* | CP |
| KY781938 | WDV-Knd2 | May-2013 | *Triticum aestivum* | CP |
| KY781939 | WDV-Kob | Jun-2016 | *Triticum aestivum* | CP |
| KY781940 | WDV-Lip | May-2015 | *Hordeum vulgare* | CP |
| KY781941 | WDV-Luk | Apr-2016 | *Hordeum vulgare* | CP |
| KY781942 | WDV-Sos | Apr-2016 | *Hordeum vulgare* | CP |
| KY781943 | WDV-Sz1 | May-2012 | *Hordeum vulgare* | CP |
| KY781944 | WDV-Sz2 | May-2012 | *Triticum aestivum* | CP |
| KY781945 | WDV-Sz3 | Apr-2013 | *Triticum aestivum* | CP |
| KY781946 | WDV-Sz4 | May-2012 | *Triticum aestivum* | CP |
| KY781947 | WDV-Wp | May-2015 | *Hordeum vulgare* | CP |
| KY781948 | WDV-Wtr | May-2015 | *Secale cereale* | CP |
| MT460909 | WDV-Strz | May-2013 | *Triticum aestivum* | CP |
| Spain | | | | |
| HF968639 | BamKl1 | Sep-2012 | *Hordeum vulgare* | MP, CP, Rep, RepA |
| HF968640 | BamKl2 | Sep-2012 | *Hordeum vulgare* | MP, CP, Rep, RepA |
| HF968641 | BamL&S | Sep-2012 | *Hordeum vulgare* | MP, CP, Rep, RepA |
| HF968642 | HindCl1 | Sep-2012 | *Hordeum vulgare* | MP, CP, Rep, RepA |
| HF968643 | HindCl6 | Sep-2012 | *Hordeum vulgare* | MP, CP, Rep, RepA |
| HF968644 | HindCl2 | Sep-2012 | *Hordeum vulgare* | MP, CP, Rep, RepA |
| HF968645 | HindCl3 | Sep-2012 | *Hordeum vulgare* | MP, CP, Rep, RepA |
| Turkey |  |  |  |  |
| AJ783960 | Hordeum vulgare | Apr-2004 | *Hordeum vulgare* | MP, CP, Rep, RepA |
| Sweden | | | | |
| AJ311031 | Enkoping1 | 1998 | *Triticum aestivum* | MP, CP, Rep, RepA |
| AM491490 | Enkoping2 | 1998 | *Triticum aestivum* | MP, CP, Rep, RepA |
| Ukraine | | | | |
| FN806783 | WDV-Uk-g | 2008 | *Triticum aestivum* | MP, CP, Rep, RepA |
| FN806784 | WDV-Uk-Miron | 2009 | *Triticum aestivum* | MP, CP, Rep, RepA |
| FN806787 | WDV-Uk-Odessa | 2009 | *Triticum aestivum* | MP, CP, Rep, RepA |
| United Kingdom | | | | |
| HF968636 | UK193WDV_ KpnKl8 | May-2012 | *Triticum aestivum* | MP, CP, Rep, RepA |
| HF968635 | UK193WDV_ KpnKl6 | May-2012 | *Triticum aestivum* | MP, CP, Rep, RepA |
| HF968634 | UK193WDV_ KpnKl5 | May-2012 | *Triticum aestivum* | MP, CP, Rep, RepA |
| HF968633 | UK193WDV_ KpnKl3 | May-2012 | *Triticum aestivum* | MP, CP, Rep, RepA |
